# The Single-Prolonged Stress Model Fails to Produce Behavioral or Corticosterone Alterations in Rats

**DOI:** 10.1523/ENEURO.0168-25.2025

**Published:** 2026-01-30

**Authors:** Moriah McGuier, Elise Bragg, Paul Holtzheimer, Wilder Doucette

**Affiliations:** ^1^ Department of Psychiatry, Dartmouth-Hitchcock Medical Center, Lebanon, New Hampshire 03766; ^2^ National Center for PTSD, White River Junction Veteran Affairs Medical Center, White River Junction, Vermont 05001; ^3^ Department of Psychiatry, Dartmouth-Hitchcock Medical Center, Geisel School of Medicine at Dartmouth, Lebanon, New Hampshire 03766

**Keywords:** anxiety-like behavior, corticosterone, HPA axis, rat, single prolonged stress model, social behavior

## Abstract

There is a critical need for robust and reliable preclinical models for posttraumatic stress disorder (PTSD) to better understand pathophysiological mechanisms and support the development of novel treatments. The single prolonged stress (SPS) model has been previously utilized to investigate various acute behavioral effects and stress hormone changes in rodents. This study paired anxiety-like and social behavioral evaluations with corticosterone assessment as a complementary physiological biomarker to determine the presence of robust and intervenable phenotypes following SPS. Sprague Dawley rats (*N* = 36, 30 male and 6 female) received SPS model induction (e.g., restraint with odorant, forced-swim, diethyl ether exposure, and isolation) or control handling. Serum corticosterone and behavioral assessments, including the open field test (OFT) and a social motivation test (SMT), were investigated at 1 and 2 weeks following SPS induction. This SPS model did not induce anxiety-like or locomotive differences assessed in the OFT (*p*'s > 0.05). Similarly, SPS did not appear to alter social preference or avoidance in the SMT (*p*'s > 0.05), as groups had similar novel social and novel object interaction levels. SPS-paired cue re-exposure did not unmask group differences in these behaviors. Corticosterone levels were also unaltered between groups in the weeks following SPS (*p* = 0.178). In the absence of other stressors or modifications, the null behavioral and corticosterone findings in the weeks following SPS suggest that this SPS protocol may not reliably produce adequately robust or intervenable phenotypes.

## Significance Statement

Having robust and intervenable preclinical models for PTSD is critical, and outcomes that resolve acutely may not relate to the long-term consequences of trauma. Further, sufficiently aversive models can be paired with a cue to enhance behavioral phenotypes or test interventions during cue re-exposure. This study evaluated the robustness and reproducibility of phenotypes in rats 1 and 2 weeks following SPS and with cue re-exposure. Anxiety-like and social behavioral changes were not recapitulated following SPS, and cue re-exposure did not enhance any phenotypes. Corticosterone was not altered 1 or 2 weeks following SPS. These null findings suggest that the SPS model, without modification for enhanced trauma induction, does not induce adequate psychological or physiological stress to reliably produce these phenotypes.

## Introduction

Preclinical, rodent models have been utilized in attempts to better understand posttraumatic stress disorder (PTSD) development, develop new therapies, and define treatment targets. PTSD, a complex and severe psychiatric disorder, significantly impacts individuals following exposure to trauma (Goldstein et al., 2016; Wisco et al., 2022). However, susceptibility and resistance factors related to PTSD remain poorly understood (Benjet et al., 2016; Skórzewska et al., 2020). Moreover, current psychotherapeutic or pharmacologic approaches may offer limited relief to symptoms experienced, including intrusive thoughts, avoidance, negative alterations to cognition and mood, arousal, and reactivity (Brady et al., 2000; Stein et al., 2003). Despite the clear need for robust preclinical models, developing or choosing a single, accepted model for translational PTSD studies remains a challenge.

One commonly reported preclinical model for PTSD is the Single Prolonged Stress (SPS) model. In the classic induction protocol for SPS, rats are exposed to a series of multi-modal stressors (i.e., restraint, forced-swim, and diethyl ether exposure for loss of consciousness) followed by a 7 d isolation window (Liberzon et al., 1997). Variations in the SPS model range from the described classic SPS protocol, SPS with a paired cue, or SPS with a modified or additional stressor (Knox et al., 2012a; Canto-de-Souza et al., 2021). Odorants have been previously paired with the restraint period of SPS induction to create a conditioned stimulus (CS; Toledano and Gisquet-Verrier, 2014), which may reinvigorate or unmask behavioral phenotypes during re-exposure. Past findings have suggested that the classic SPS model in rats produces both behavioral (i.e., anxiety-like, hyperarousal, social interaction changes) and physiological changes (Yamamoto et al., 2009; Souza et al., 2017; Lisieski et al., 2018; Ferland-Beckham et al., 2021).

However, SPS outcomes may be temporally limited and sensitive to procedural differences, leading to variable findings. The reported timing of behaviors following SPS varies, with most studies focusing on acute effects in the days following model induction and some reports 2–4 weeks later (Wu et al., 2016; Mancini et al., 2021). Key behavioral phenotypes that have been evaluated following SPS are anxiety and depressive-like outcomes, social behaviors, and cognitive impairments (Lisieski et al., 2018). The open field test (OFT) is a common assay of anxiety-like and exploratory behaviors in rodents (Walsh and Cummins, 1976). Some groups have reported anxiety-like behavioral phenotypes (Wu et al., 2016; Iqbal et al., 2024), while others failed to recapitulate the phenotypes (Harvey et al., 2006; Eagle et al., 2013b; Lisieski and Perrine, 2017; Wu et al., 2017). Individuals with PTSD have also been reported to have changes in sociability, including increased avoidance (Charuvastra and Cloitre, 2008; Janssen et al., 2022). Some studies have shown altered social interactions, avoidance, and contextual fear following SPS model induction (Kohda et al., 2007; Eagle et al., 2013a), indicating potential social and motivational dysregulation.

In humans, the glucocorticoid cortisol has been shown to play a critical role in the body's stress response, similar to corticosterone (CORT) in rodents (Souza et al., 2017). The hypothalamic-pituitary-adrenal (HPA) axis release of glucocorticoids has been reported to be dysregulated in individuals with PTSD (Speer et al., 2019; Danan et al., 2021). Rodent studies have shown that SPS may lead to dysregulated CORT (Liberzon et al., 1997; Knox et al., 2012a,b; Toledano et al., 2013; Verbitsky et al., 2020), although timing and effects differ between studies. Physiological changes noted in individuals with PTSD should be reflected in reliable preclinical models and provide a complementary biomarker to behavioral or therapeutic evaluation.

This study aimed to provide a multilevel readout of stress-related behavioral and corticosterone changes to evaluate the robustness and reproducibility of the SPS model in rats. We hypothesized that SPS model induction would result in increased anxiety-like and social avoidant behaviors as previously reported and that trauma cue re-exposure would enhance behavioral phenotypes between groups. We further hypothesized that serum CORT levels would be dysregulated when sampled 1 week following SPS.

## Materials and Methods

### Animal use

Sprague Dawley rats (*N* = 36, 30M, 6F) were purchased from Charles River Laboratories at 60 d of age and were individually housed on a reverse 12 h light cycle with *ad libitum* food and water unless otherwise specified. Experiments began at 10 weeks of age (∼70 d). All experiments for this study were conducted in accordance with the National Institute of Health Guide for the Care and Use of Laboratory Animals and were approved by the Institutional Animal Care and Use Committee of Dartmouth College.

### Experimental timeline

**Figure 1.**
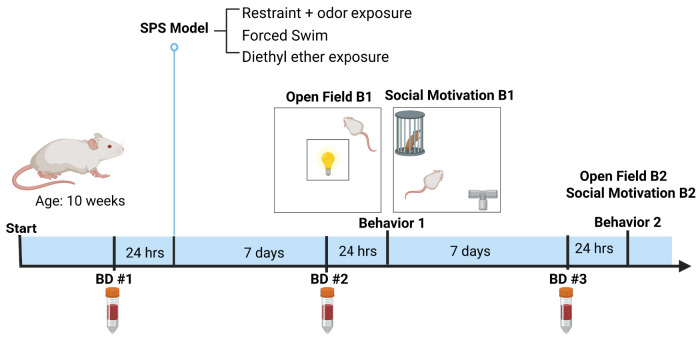
Timeline of experimental methodology for the single prolonged stress model and assessments. *N* = 36 rats received either SPS induction or control handling, where SPS induction consisted of the multimodal stressor sequence including 2 h. restraint and odorant exposure followed by forced swim (15 min) and diethyl ether exposure. In addition, three lateral tail vein blood draws were collected (BD1, BD2, BD3) and two behavioral timepoints at 1 (B1) and 2 weeks (B2) post-SPS induction were assessed. Behaviors investigated at these timepoints were the open field test and social motivation test.

Male and female rats (*N* = 36), received in three cohorts, were acclimated (pair-housed) for 1 week after arrival prior to collection of a baseline serum sample via lateral tail vein blood collection. Cohorts were counterbalanced for SPS or control group sample sizes. Twenty-four hours following the baseline blood collection, rats were then exposed to the SPS model (restraint + odorant, forced swim, and diethyl ether exposure) or control handling with odor exposure (Fig. 1). Further behavioral testing and blood sampling occurred 1 and 2 weeks following induction of the SPS model. Following the experimental conclusion, rats were humanely killed.

### Single prolonged stress model

A total of *N* = 36 rats were either exposed to the components of the SPS model or were handled as controls. SPS induction is a multistressor sequence involving 2 h of restraint via decapiCone paired with odorant exposure of 20% lemon oil in mineral oil, individual forced-swim (15 min), and diethyl ether exposure until loss of consciousness (10 ml, <5–10 min). The restraint portion of SPS was paired with odor exposure, and control animals were exposed to the odorant for 2 h in their home cage after handling. All stressors were done individually in SPS animals; no stressors were conducted in a group context. All animals were closely monitored for health concerns during SPS and were allowed to recover for 5–10 min between stressors. After diethyl ether recovery, rats were returned to clean single-housed cages, and rats were isolated for a week (no handling or housekeeping from lab or animal care staff). At each stressor step, control animals were handled to simulate the operator transfer and handling.

### Serum corticosterone collection and analysis

To collect serum for corticosterone assessment, lateral tail vein blood samples were collected at experimental timepoints BD1, BD2, and BD3 (pre-SPS, 1 week, and 2 weeks post-SPS). Baseline (BD1) was collected 24 h prior to the SPS model, as a preinduction comparison of CORT levels in both groups and to assess the variation in groups. The second and third samplings (BD2, BD3) were chosen to capture the CORT levels immediately following the week of isolation and 2 weeks following SPS induction. BD2 and BD3 serum samplings were performed 24 h before behavioral testing for temporal comparison of behavioral and CORT changes. To achieve this, rats were briefly anesthetized using 2–3% isoflurane, and tails were warmed via a heating pad before utilizing a heparinized butterfly catheter to gather 200–300 µl whole blood. Brief isoflurane exposure allowed rapid sampling without pain or distress and uniformity of sampling across subjects and repeated collections. Samples were collected starting ∼1 h before the end of their light phase, and each sampling was 5–7 min from start to finish. After sampling, animals were returned to their home cage. Whole blood samples were transferred to a collection tube and clotted at room temperature for 45 min before being held on ice until further processing. Samples were centrifuged for 15 min at 1,960 × *g* at 4°C. Supernatant was collected, aliquoted, and stored at −80°C until ready for use. To provide quantification of corticosterone in each sample, ELISA kits (Bio-Techne, R&D Systems, KGE009) were run with duplicate samples and appropriate standards. To control batch differences among kits and between cohorts, sample results were normalized to the standard curve of their kit before being aggregated.

### Open field test

OFT was used to assess general locomotion, exploration, and anxiety-like behaviors following SPS induction. The apparatus for this task was a 60 cm × 60 cm × 40 cm arena, with walls and floors painted black for contrast and lighting. An overhead light was concentrated on the center one-third of the arena, providing center lighting (350 lumens). Light around the arena edges was <200 lumens, providing an anxiogenic center field. At timepoints B1 and B2 (i.e., 1 and 2 weeks following SPS), rats were placed individually in the arena for 15 min by the operator. Behavior recognition software (Noldus EthoVision XT) was used for tracking and locomotion of animals, as well as scoring various center-based metrics. Behavioral metrics from the OFT [i.e., velocity (cm/s), percent time in the center zone] were calculated for statistical comparison across time bins in B1 and B2. The sessions were sectioned into five 3 min time bins during analysis for comparison of behavioral outputs between groups and behavioral changes throughout the duration of the session.

### Social motivation test

Social motivation test (SMT) was employed to test motivation or avoidance toward novel social and novel object interactions. The apparatus for this task was a 60 cm × 60 cm × 40 cm arena, with walls and floors painted black for contrast and lighting. The lighting condition for this test was low lighting, with only minimal diffuse light (<100 lumens). The novel social rat was a sex and age-matched conspecific, and different novel animals were used for behavior tests 1 and 2 weeks post-SPS. Two visually and texturally different novel objects were used for the two SMT timepoints, B1 and B2 (1, 2 weeks post-SPS). The object for B1 (blue PVC pipe fitting, 4 in length, three-way pipe) and the object for B2 (1.0 in white PVC ball valve, 3.8 in length) differed by behavioral timepoint, but the location in the arena was consistent. Novel social rats and novel objects were placed on opposing corners of the arena, and rats were placed in the center of the arena before freely behaving for 15 min. Behavioral metrics from this task were again determined through video analysis. These metrics from the SMT (i.e., percent time in novel object zone, percent time in novel social zone) were calculated across time bins in B1 and B2 (five 3 min time bins).

### SPS-paired cue re-exposure during behavior

All cohorts were characterized for behavioral metrics at B1 and B2 (*N* = 12 SPS, *N* = 12 Ctrl) as described. Cohort 3 [*N* = 6 SPS (3M, 3F), *N* = 6 Ctrl (3M, 3F)] experienced re-exposure of the SPS-paired cue during OFT and SMT as a CS associated with model induction stress. Five milliliters of the odorant were placed on a cotton pad attached to the arena wall for each animal after arena cleaning and was left for the duration of each OFT and SMT behavioral session as an SPS trauma cue.

### Statistical analysis

The analysis plan for these measures includes the statistical approach for behavioral and physiological outcomes, differential analysis of cohorts based on condition, and assessment of variation within groups. Following ELISA quantification of absorbance values (450 nm), the concentration of CORT (ng/mL) was determined through interpolation of absorbance values determined by within-kit standard curve, using a sigmoidal four-parameter fit. Statistical analysis of normalized CORT values for group differences at 1 and 2 weeks post-SPS between groups was performed using a one-way ANOVA and Bonferroni’s multiple-comparison correction. Additionally, the percent change in CORT from baseline to 1 and 2 weeks post-SPS was statistically assessed via multiple unpaired *t* tests. Following video analysis using behavioral recognition software, time-binned values for behavioral metrics from OFT and SMT were statistically compared with separate two-way ANOVAs for behavioral timepoints B1 and B2. A combined analysis was performed for cohorts 1 and 2 (i.e., *N* = 12 per group, males), and a separate analysis was performed for cohort 3 (i.e., *N* = 6 (3 M, 3F) per group) to differentiate the effects of behavioral outcomes of cue re-exposure. There was not a high enough sample size of females to perform a sex-specific analysis or to include sex as a cofactor (i.e., in a mixed effects analysis), so males and females of cohort 3 were combined within SPS and Ctrl conditions. To address individual variation within groups across assessments, *Z*-scores from the Ctrl group mean were determined for each animal (e.g., OFT percent time in center zone, SMT percent time in social zone, % change from BD1-BD2, BD1-BD3). For a more comprehensive review of statistical analysis approaches and outcomes in this study, see Table 1. For statistical analysis and figure creation, we use Prism (v. 10.2.1, GraphPad) and BioRender (BioRender.com).

**Table 1.** Detailed statistical table

Fig	Comparison	Type of test	Statistic	95% CI
2*a*	Velocity (cm/s), B1, OFT time bins in SPS and Ctrl rats	Two-way ANOVA	Group: *F*_(1,22)_ = 4.286	*p* = 0.0504
2*a*	Velocity (cm/s), B1, OFT time bins in SPS and Ctrl rats	Two-way ANOVA	Time: *F*_(3.147,69.24)_ = 12.02	*p* = 0.0001
2*a*	Velocity (cm/s), B1, OFT time bins in SPS and Ctrl rats	Two-way ANOVA	Group × time: *F*_(4,88)_ = 0.9203	*p* = 0.4559
2*b*	Velocity (cm/s), B2, OFT time bins in SPS and Ctrl rats	Two-way ANOVA	Group: *F*_(1,22)_ = 0.899	*p* = 0.3533
2*b*	Velocity (cm/s), B2, OFT time bins in SPS and Ctrl rats	Two-way ANOVA	Time: *F*_(3.240,71.29)_ = 7.895	*p* = 0.0001
2*b*	Velocity (cm/s), B2, OFT time bins in SPS and Ctrl rats	Two-way ANOVA	Group × time: *F*_(4,88)_ = 0.8290	*p* = 0.5102
2*c*	Time (%) in center zone, B1, OFT time bins in SPS and Ctrl rats	Two-way ANOVA	Group: *F*_(1,22)_ = 0.4213	*p* = 0.5230
2*c*	Time (%) in center zone, B1, OFT time bins in SPS and Ctrl rats	Two-way ANOVA	Time: *F*_(2.738,60.23)_ = 2.347	*p* = 0.0870
2*c*	Time (%) in center zone, B1, OFT time bins in SPS and Ctrl rats	Two-way ANOVA	Group × time: *F*_(4,88)_ = 0.4490	*p* = 0.7729
2*d*	Time (%) in center zone, B2, OFT time bins in SPS and Ctrl rats	Two-way ANOVA	Group: *F*_(1,22)_ = 0.5910	*p* = 0.4502
2*d*	Time (%) in center zone, B2, OFT time bins in SPS and Ctrl rats	Two-way ANOVA	Time: *F*_(2.136,46.99)_ = 2.243	*p* = 0.1141
2*d*	Time (%) in center zone, B2, OFT time bins in SPS and Ctrl rats	Two-way ANOVA	Group × time: *F*_(4,88)_ = 0.9808	*p* = 0.4223
2*e*	Time (%) in object zone, B1, SMT time bins in SPS and Ctrl rats	Two-way ANOVA	Group: *F*_(1,22)_ = 0.4412	*p* = 0.5134
2*e*	Time (%) in object zone, B1, SMT time bins in SPS and Ctrl rats	Two-way ANOVA	Time: *F*_(3,65.99)_ = 1.720	*p* = 0.1713
2*e*	Time (%) in object zone, B1, SMT time bins in SPS and Ctrl rats	Two-way ANOVA	Group × time: *F*_(4,88)_ = 0.8620	*p* = 0.4901
2*f*	Time (%) in object zone, B2, SMT time bins in SPS and Ctrl rats	Two-way ANOVA	Group: *F*_(1,22)_ = 0.4028	*p* = 0.5322
2*f*	Time (%) in object zone, B2, SMT time bins in SPS and Ctrl rats	Two-way ANOVA	Time: *F*_(2.916,64.15)_ = 3.167	*p* = 0.0315
2*f*	Time (%) in object zone, B2, SMT time bins in SPS and Ctrl rats	Two-way ANOVA	Group × time: *F*_(4,88)_ = 0.4716	*p* = 0.7564
2*g*	Time (%) in social zone, B1, SMT time bins in SPS and Ctrl rats	Two-way ANOVA	Group: *F*_(1,22)_ = 0.1374	*p* = 0.7144
2*g*	Time (%) in social zone, B1, SMT time bins in SPS and Ctrl rats	Two-way ANOVA	Time: *F*_(3.190,70.18)_ = 3.957	*p* = 0.0101
2*g*	Time (%) in social zone, B1, SMT time bins in SPS and Ctrl rats	Two-way ANOVA	Group × time: *F*_(4,88)_ = 0.5001	*p* = 0.7357
2*h*	Time (%) in social zone, B2, SMT time bins in SPS and Ctrl rats	Two-way ANOVA	Group: *F*_(1,22)_ = 1.754	*p* = 0.1990
2*h*	Time (%) in social zone, B2, SMT time bins in SPS and Ctrl rats	Two-way ANOVA	Time: *F*_(3.236,71.18)_ = 1.533	*p* = 0.2106
2*h*	Time (%) in social zone, B2, SMT time bins in SPS and Ctrl rats	Two-way ANOVA	Group × time: *F*_(4,88)_ = 1.096	*p* = 0.3637
3*a*	Velocity (cm/s) with cue re-exposure, B1, OFT time bins in SPS and Ctrl rats	Two-way ANOVA	Group: *F*_(1,10)_ = 0.2608	*p* = 0.6206
3*a*	Velocity (cm/s) with cue re-exposure, B1, OFT time bins in SPS and Ctrl rats	Two-way ANOVA	Time: *F*_(2.215,22.15)_ = 15.21	*p* = 0.0001
3*a*	Velocity (cm/s) with cue re-exposure, B1, OFT time bins in SPS and Ctrl rats	Two-way ANOVA	Group × time: *F*_(4,40)_ = 0.6494	*p* = 0.6307
3*b*	Velocity (cm/s) with cue re-exposure, B2, OFT time bins in SPS and Ctrl rats	Two-way ANOVA	Group: *F*_(1,10)_ = 0.05611	*p* = 0.8175
3*b*	Velocity (cm/s) with cue re-exposure, B2, OFT time bins in SPS and Ctrl rats	Two-way ANOVA	Time: *F*_(2.031, 20.31)_ = 20.89	*p* = 0.0001
3*b*	Velocity (cm/s) with cue re-exposure, B2, OFT time bins in SPS and Ctrl rats	Two-way ANOVA	Group × time: *F*_(4,40)_ = 1.377	*p* = 0.2591
3*c*	Time (%) in center zone (cue re-exposure), B1, OFT time bins in SPS and Ctrl rats	Two-way ANOVA	Group: *F*_(1,10)_ = 0.0730	*p* = 0.7924
3*c*	Time (%) in center zone (cue re-exposure), B1, OFT time bins in SPS and Ctrl rats	Two-way ANOVA	Time: *F*_(2.9,29)_ = 1.629	*p* = 0.2052
3*c*	Time (%) in center zone (cue re-exposure), B1, OFT time bins in SPS and Ctrl rats	Two-way ANOVA	Group × time: *F*_(4,40)_ = 1.023	*p* = 0.4070
3*d*	Time (%) in center zone (cue re-exposure), B1, OFT time bins in SPS and Ctrl rats	Two-way ANOVA	Group: *F*_(1,10)_ = 0.086	*p* = 0.7749
3*d*	Time (%) in center zone (cue re-exposure), B1, OFT time bins in SPS and Ctrl rats	Two-way ANOVA	Time: *F*_(2.059,20.59)_ = 2.019	*p* = 0.1574
3*d*	Time (%) in center zone (cue re-exposure), B1, OFT time bins in SPS and Ctrl rats	Two-way ANOVA	Group × time: *F*_(4,40)_ = 1.301	*p* = 0.2861
3*e*	Time (%) in object zone (cue re-exposure), B1, SMT time bins in SPS and Ctrl rats	Two-way ANOVA	Group: *F*_(1,10)_ = 0.5165	*p* = 0.4888
3*e*	Time (%) in object zone (cue re-exposure), B1, SMT time bins in SPS and Ctrl rats	Two-way ANOVA	Time: *F*_(2.274,22.74)_ = 3.319	*p* = 0.0490
3*e*	Time (%) in object zone (cue re-exposure), B1, SMT time bins in SPS and Ctrl rats	Two-way ANOVA	Group × time: *F*_(4,40)_ = 0.4447	*p* = 0.7756
3*f*	Time (%) in object zone (cue re-exposures), B2, SMT time bins in SPS and Ctrl rats	Two-way ANOVA	Group: *F*_(1,10)_ = 0.4575	*p* = 0.5141
3*f*	Time (%) in object zone (cue re-exposures), B2, SMT time bins in SPS and Ctrl rats	Two-way ANOVA	Time: *F*_(3.073,30.73)_ = 0.5013	*p* = 0.6885
3*f*	Time (%) in object zone (cue re-exposures), B2, SMT time bins in SPS and Ctrl rats	Two-way ANOVA	Group × time: *F*_(4,40)_ = 0.9342	*p* = 0.4539
3*g*	Time (%) in social zone (cue re-exposures), B1, SMT time bins in SPS and Ctrl rats	Two-way ANOVA	Group: *F*_(1,10)_ = 0.6011	*p* = 0.4561
3*g*	Time (%) in social zone (cue re-exposures), B1, SMT time bins in SPS and Ctrl rats	Two-way ANOVA	Time: *F*_(1.821,18.21)_ = 0.7636	*p* = 0.4689
3*g*	Time (%) in social zone (cue re-exposures), B1, SMT time bins in SPS and Ctrl rats	Two-way ANOVA	Group × time: *F*_(4,40)_ = 0.7680	*p* = 0.5524
3*h*	Time (%) in social zone (cue re-exposures), B2, SMT time bins in SPS and Ctrl rats	Two-way ANOVA	Group: *F*_(1,10)_ = 2.669	*p* = 0.1334
3*h*	Time (%) in social zone (cue re-exposures), B2, SMT time bins in SPS and Ctrl rats	Two-way ANOVA	Time: *F*_(2.626,26.2)_ = 3.412	*p* = 0.0371
3*h*	Time (%) in social zone (cue re-exposures), B2, SMT time bins in SPS and Ctrl rats	Two-way ANOVA	Group × time: *F*_(4,40)_ = 1.352	*p* = 0.2679
4*a*	Normalized CORT levels (ng/ml), BD1-BD3, SPS, and Ctrl rats across cohorts	One-way ANOVA	Group: *F*_(5,101)_ = 1.56	*p* = 0.1778

Comprehensive report of statistical analyses performed.

## Results

*N* = 18 rats were exposed to the SPS model, and *N* = 18 were tested as paired controls through 3 cohorts. Blood sampling was not possible for one animal, resulting in an *N* = 17 sample size for SPS CORT metrics. Results for cohorts that were not re-exposed to the cue during behavior [cohorts 1 and 2, *N* = 12 (males) per group] and the cohort with cue re-exposure [cohort 3, *N* = 6 (3M, 3F) per group] are separated for behavioral comparison.

### Effects of the SPS model on anxiety-like and social behavior in rats

#### OFT outcomes

**Figure 2.**
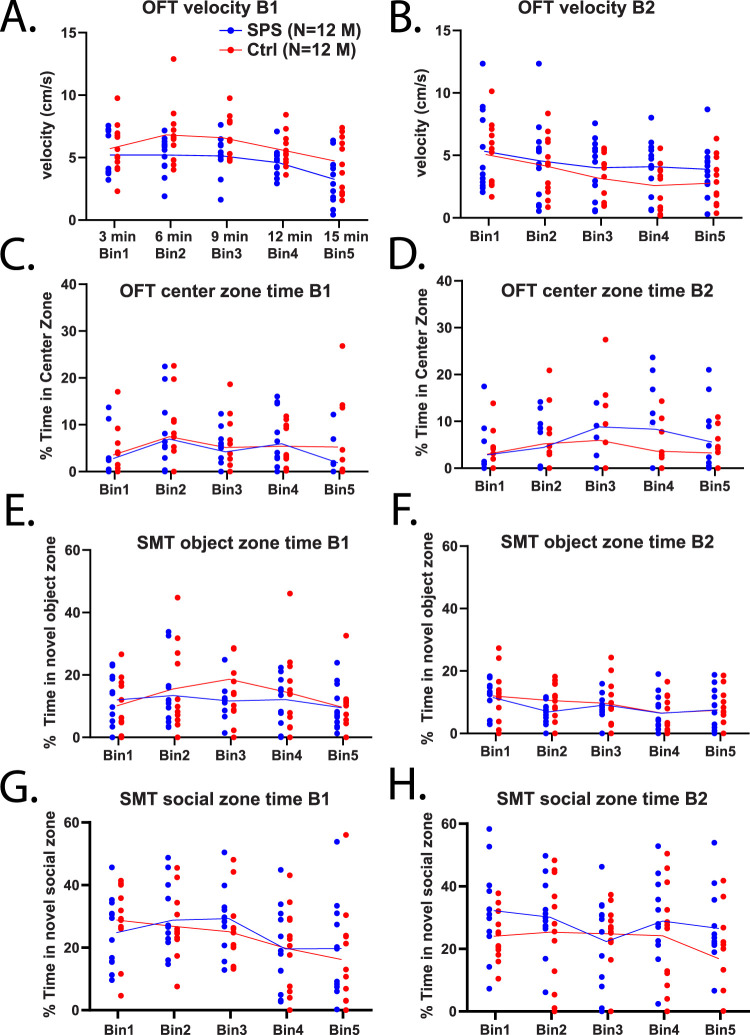
Assessment of behavioral changes following SPS in the OFT and SMT. *N* = 12 SPS (blue) and *N* = 12 control (red) rats had 15 min OFT and SMT sessions. OFT and SMT were tested at 1 week post-SPS (B1) and 2 weeks post-SPS (B2). Results for each behavior is displayed per 3 min bin. Each bin is the average value over 3 min per animal. General locomotion, exploratory and anxiety-like behaviors were explored in the OFT. Average velocity of groups during the OFT at timepoints B1 (***A***) and B2 (***B***). The percent time spent in the center zone of OFT while freely exploring the arena was determined for B1 (***C***) and B2 (***D***) timepoints per group. SMT was utilized to assess motivation or avoidance toward novel object or social experiences, and rats had free access to both over 15 min. Metrics for interaction in the SMT were determined, % time in the novel object zone is shown per bin at B1 (***E***) and B2 (***F***). Interactions in the novel social zone, shown as % time for B1 (***G***) and B2 (***H***).

The main metrics of OFT are velocity for locomotion and arena center-based metrics to evaluate anxiety-like behaviors. These metrics were assessed per 3 min time bins for group-based comparisons and for potential behavioral changes through time in the task. Velocity (cm/s) during the OFT had a significant effect of time across bins revealed via two-way ANOVA in B1 (Fig. 2*A*; *F*_(3.147,69.24)_ = 12.02, *p* = 0.0001) and B2 (Fig. 2*B*; *F*_(3.240,71.29)_ = 7.895, *p* = 0.0001), as velocity decreased through the five time bins. No significant group effect (*F*_(1,22)_ = 4.286, *p* = 0.0504) or group–time interaction effect (*F*_(4,88)_ = 0.9203, *p* = 0.4559) was revealed in B1, similarly no group (*F*_(1,22)_ = 0.899, *p* = 0.3533) or group × time effect (*F*_(4,88)_ = 0.8290, *p* = 0.5102) was revealed in B2. SPS and control rats were evaluated for percent time spent in the center zone during the OFT, and no group differences (*F*_(1,22)_ = 0.4213, *p* = 0.5230), effect of time (*F*_(2.738,60.23)_ = 2.347, *p* = 0.0870), or group × time effect (*F*_(4,88)_ = 0.4490, *p* = 0.7729) were found in B1 (Fig. 2*C*). This was also true when tested at timepoint B2, with no effect of group (*F*_(1,22)_ = 0.5910, *p* = 0.4502), time (*F*_(2.136,46.99)_ = 2.243, *p* = 0.1141), or their interaction (*F*_(4,88)_ = 0.9808, *p* = 0.4223) revealed (Fig. 2*D*). No significant differences in distance traveled, center zone frequency, or rearing were revealed by two-way ANOVA (data not shown; *p*'s > 0.05).

#### SMT outcomes

The main metrics of this task are the percent of time per 3 min bin spent in the novel object or novel social zones to assess motivation or avoidance of these interactions. When comparing the percent of time spent in the novel object zone at timepoint B1 (Fig. 2*E*), no group differences were revealed including no effect of group (*F*_(1,22)_ = 0.4412, *p* = 0.5134), effect of time through bins (*F*_(3,65.99)_ = 1.720, *p* = 0.1713), or the group × time interaction effect (*F*_(4,88)_ = 0.8620, *p* = 0.4901). This lack of group differences remained true in B2 (Fig. 2*F*) with no effect of group (*F*_(1,22)_ = 0.4028, *p* = 0.5322) or the interaction of group and time (*F*_(4,88)_ = 0.4716, *p* = 0.7564), although a significant effect of time across was revealed (*F*_(2.916,64.15)_ = 3.167, *p* = 0.0315) showing groups spent less time in the novel object zone across time bins. Time spent in the novel social rat zone was also compared between groups; in B1 (Fig. 2*G*) no significant differences in this metric were found between SPS and control groups with two-way ANOVA revealing no effect of group (*F*_(1,22)_ = 0.1374, *p* = 0.7144) or interaction of group × time (*F*_(4,88)_ = 0.5001, *p* = 0.7357) but a significant effect of time (*F*_(3.190,70.18)_ = 3.957, *p* = 0.0101) as both groups spent less time in the social zone through time bins. In the second behavioral timepoint tested, B2 (Fig. 2*H*), there were again no group differences in time spent with in the novel social zone, with two-way ANOVA revealing no effect of group (*F*_(1,22)_ = 1.754, *p* = 0.1990), time (*F*_(3.236,71.18)_ = 1.533, *p* = 0.2106), or group × time through bins evaluated (*F*_(4,88)_ = 1.096, *p* = 0.3637). Rats of both groups spent more time on average in the novel social zone than in the novel object zone during both timepoints. No significant difference in distance traveled, object or social zone frequencies, or zone alterations were revealed by two-way ANOVA (data not shown; *p*'s > 0.05).

### Behavioral results during SPS-paired cue re-exposure

**Figure 3.**
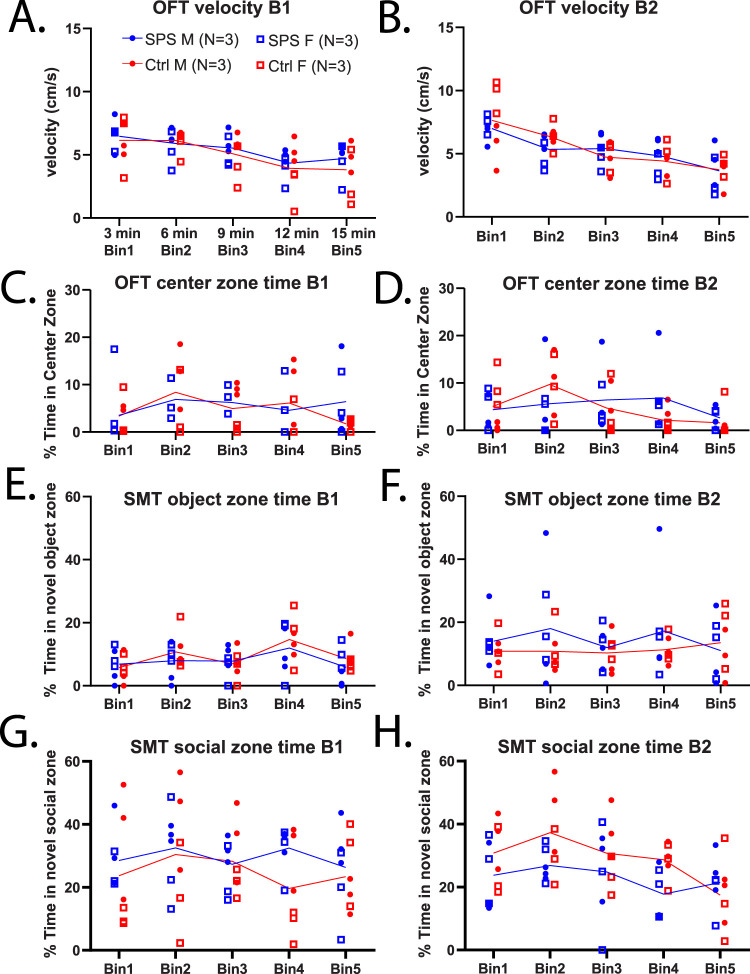
Assessment of behavioral changes following SPS in the OFT and SMT with paired cue re-exposure (cohort 3). SPS (blue) and control (red) rats were in the OFT and SMT each for 15 min, and the cue (odor) was placed in the arena for both tasks. Results for each behavior are displayed per 3 min bin. Each bin is the average value over 3 min per animal. OFT and SMT were tested at 1 week post-SPS (B1) and 2 weeks post-SPS (B2) as in Figure 2 results, but with the addition of cue re-exposure during behavioral testing. General locomotion, exploratory, and anxiety-like behaviors were explored in the OFT. Average velocity of groups during the OFT at timepoints B1 (***A***) and B2 (***B***). The percent time spent in the center zone of OFT while freely exploring the arena was determined for B1 (***C***) and B2 (***D***) timepoints per group. SMT was utilized to assess motivation or avoidance toward novel object or social experiences, and rats had free access to both over 15 min. Metrics for interaction in the SMT were determined, % time in the novel object zone is shown per bin at B1 (***E***) and B2 (***F***). Interactions in the novel social zone, shown as % time for B1 (***G***) and B2 (***H***).

The effect of re-exposure to the SPS-paired cue during OFT and SMT was determined for SPS and control rats in cohort 3 at behavioral timepoints B1 and B2 across 3 min time bins (Fig. 3).

#### OFT outcomes with cue re-exposures

Evaluation of locomotion and center-based metrics during cue re-exposure in OFT. When assessing velocity differences in B1 (Fig. 3*A*), a two-way ANOVA did not reveal a group effect (*F*_(1,10)_ = 0.2608, *p* = 0.6206), but an effect of time was discovered (*F*_(2.215,22.15)_ = 15.21, *p* = 0.0001). Similarly, in the second OFT timepoint, B2 (Fig. 3*B*), velocity did not differ between groups (*F*_(1,10)_ = 0.05611, *p* = 0.8175) but a significant effect of time was revealed (*F*_(2.031, 20.31)_ = 20.89, *p* = 0.0001). No group × time effects were revealed during B1 (*F*_(4,40)_ = 0.6494, *p* = 0.6307) or B2 (*F*_(4,40)_ = 1.377, *p* = 0.2591). Groups were additionally assessed for percent time in the center zone of OFT with no significant differences found during B1 (Fig. 3*C*), including effect of group (*F*_(1,10)_ = 0.0730, *p* = 0.7924), time through bins (*F*_(2.9,29)_ = 1.629, *p* = 0.2052), or group × time (*F*_(4,40)_ = 1.023, *p* = 0.407). Similarly, in B2 (Fig. 3*D*), no significant effect was revealed for group (*F*_(1,10)_ = 0.086, *p* = 0.7749), time (*F*_(2.059,20.59)_ = 2.019, *p* = 0.1574), or group × time (*F*_(4,40)_ = 1.301, *p* = 0.2861) via two-way ANOVA. SPS and control performances were similar across timepoints and to individuals who did not experience cue re-exposure. No significant differences in distance traveled, center zone frequency, or rearing were revealed by two-way ANOVA (data not shown; *p*'s > 0.05) during OFT with cue re-exposure.

#### SMT outcomes with cue re-exposures

Novel object and novel social interactions were not altered by cue re-exposure in SPS rats. While assessing percent time in the novel object zone during cue re-exposure, no effects of group (*F*_(1,10)_ = 0.5165, *p* = 0.4888) or group × time effect (*F*_(4,40)_ = 0.4447, *p* = 0.7756) but a significant effect of time (*F*_(2.274,22.74)_ = 3.319, *p* = 0.0490) in B1 was found (Fig. 3*E*). No significant differences in percent time in the novel object zone, including group effect (*F*_(1,10)_ = 0.4575, *p* = 0.5141), effect of time (*F*_(3.073,30.73)_ = 0.5013, *p* = 0.6885), or group × time effect (*F*_(4,40)_ = 0.9342, *p* = 0.4539) occurred in B2 (Fig. 3*F*). Finally, cue re-exposure did not produce group differences in percent time in the novel social rat zone, with no effect of group (*F*_(1,10)_ = 0.6011, *p* = 0.4561), time through bins (*F*_(1.821,18.21)_ = 0.7636, *p* = 0.4689), or group × time effect (*F*_(4,40)_ = 0.7680, *p* = 0.5524) in B1 (Fig. 3*G*). In B2 (Fig. 3*H*), only a significant effect of time (*F*_(2.626,26.2)_ = 3.412, *p* = 0.0371) was revealed, with no group (*F*_(1,10)_ = 2.669, *p* = 0.1334) or group × time effects (*F*_(4,40)_ = 1.352, *p* = 0.2679). During cue re-exposure, both groups of rats spent a greater amount of time in the social zone than the object zone, with similar preferences to rat behavior without cues (Fig. 2). No significant differences in other metrics including distance traveled, object or social zone frequencies, or zone alterations were revealed by two-way ANOVA (data not shown; *p*'s > 0.05) during the SMT with cue re-exposure.

### Serum corticosterone evaluation following SPS in rats

**Figure 4.**
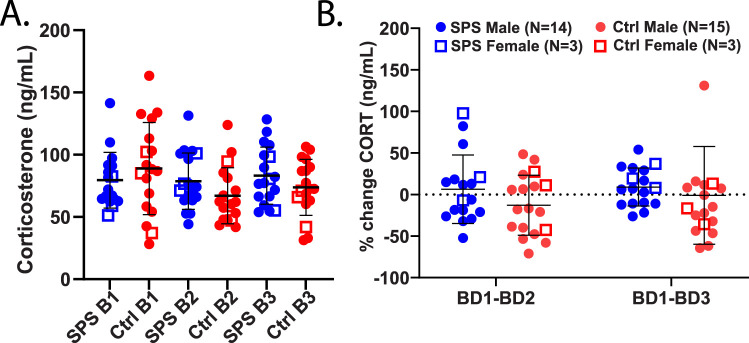
Assessment of corticosterone following SPS induction. ***A***, Normalized corticosterone values for SPS and control rats, assessed via ELISA kit in three cohorts. Sample sizes were *N* = 18 for all groups, in the exception of *N* = 17 SPS at timepoint BD1. CORT (ng/ml) is shown for both groups across timepoints from baseline (BD1) to 1 week (BD2) and 2 weeks (BD3) following SPS induction. ***B***, Within animal percent change in CORT from baseline to BD2 and BD3, where individual percentage changes are shown for both SPS (red) and control (blue) rats.

Serum samples collected at three timepoints (BD1, BD2, BD3) were processed, and quantification of corticosterone (ng/ml) was determined for duplicates of each sample. Normalized CORT values (ng/ml) were compared between SPS and Ctrl groups across timepoints (Fig. 4*A*). No group differences in CORT were revealed at 1 or 2 week timepoints following SPS via one-way ANOVA (*F*_(5, 101)_ = 1.56, *p* = 0.1778), and no significant differences were revealed after Bonferroni’s multiple comparisons. Corticosterone value averages were similar between cohorts. The percent change in CORT from baseline to 1 and 2 weeks post-SPS in both groups was also similar, with high variation in within-animal changes over time (Fig. 4*B*). Multiple unpaired *t* tests of % changes did not reveal any group differences in the BD1-BD2 (*p* = 0.1505) or BD1-BD3 (*p* = 0.5149) comparisons.

### Assessment of individual rat behavioral and corticosterone variation

**Figure 5.**
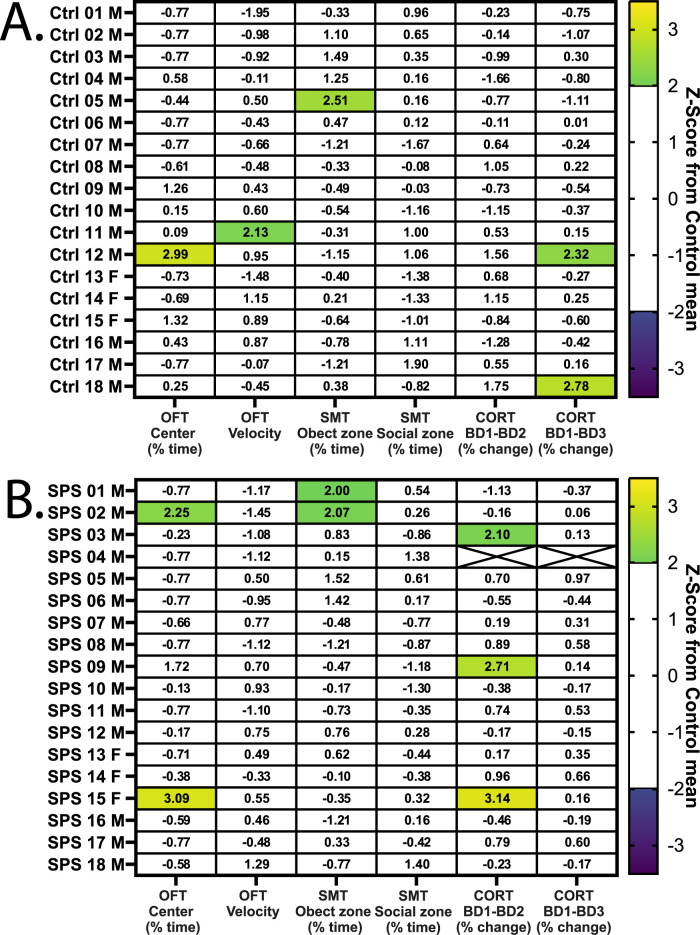
Individual and group variation across OFT and SMT behavioral and CORT phenotypes. Behavior comparison includes metrics from timepoint B1, tested 1 week following SPS. Comparisons for all metrics include the individual rat performance *Z*-scored to the mean of the control group to statistically test if the metric is significantly different than the control population (|*Z*-score >2|), to provide a multimetric comparison per rat and identify potential subgroups. ***A***, Control (*N* = 18) rat individual variation across behavioral and CORT-related metrics, in *Z*-score to control mean. ***B***, SPS (*N* = 18) rat individual and group variation across metrics, shown as *Z*-score to the control mean. *N* = 1 rat was excluded for CORT metrics due to missing sample at the pre-SPS blood draw (BD1).

To determine the level of variation across groups, individual performances across behavioral metrics and CORT measurements were *Z*-scored to the control group performance for Ctrl (Fig. 5*A*) and SPS rats (Fig. 5*B*). A low number of comparisons in either group were significantly different from the control group mean (|*Z*-score > 2|), with 7 in SPS and 5 in controls. Approximately 5% of *z*-scores reach significance, which is expected by chance given the number of comparisons. No subgroups (i.e., susceptible, resilient) in behavioral or physiological outcomes were revealed.

## Discussion

The expected behavioral and physiological changes following the SPS model in rats were not recapitulated in this study. SPS and control rats displayed similar behavior in the OFT and preferences for the novel object and novel social interaction in the SMT. These metrics were not modulated by re-exposure to the odorant previously paired with restraint during SPS. Serum CORT levels, assessed as a physiological biomarker of stress in the model, were not altered at either the 1 or 2 week timepoint. The null behavioral findings, coupled with the lack of CORT changes, suggest that published SPS models may fail to produce reliable phenotypes. Without a robust and repeatable effect in established behavioral and physiological outcomes, the usefulness of this SPS protocol is diminished. Many groups have already moved to alternate or modified SPS protocols, while still reporting them as “SPS,” highlighting the lack of robust or replicable outcomes across research groups.

### No behavioral deficits in OFT or SMT 1 or 2 weeks following SPS

Under the conditions tested, the SPS model did not produce the anxiety-like phenotype at the chosen timepoints in OFT. This differs from reported findings at 1 week post-SPS (Souza et al., 2017; Sarapultsev et al., 2024) but aligns with groups that failed to recapitulate anxiety-like phenotypes (Harvey et al., 2006; Eagle et al., 2013b; Lisieski and Perrine, 2017; Wu et al., 2017). Other studies have employed additional or alternative anxiety-related tasks (Walf and Frye, 2007), which may produce different results. Both groups spent a low percentage of time in the center zone, which may suggest a possible floor effect in exploratory behavior.

The SPS model did not significantly alter social or object-related interaction or induce avoidance in the SMT at 1 or 2 weeks. It was believed that a decrease in social interactions post-SPS would relate to socially avoidant behavior, while increased preference for social versus object novelty may lean toward a compensatory increase in social motivation. However, this lack of change or difference between groups suggests that SPS exposure did not alter social phenotypes in these metrics. It is also possible that the timepoints or the SMT are not sensitive to potential social or motivational changes.

### No effect of SPS cue re-exposure in behavioral assessments

Previous studies have found that trauma-exposed animals had greater avoidance of an odorant CS in behaviors (Toledano and Gisquet-Verrier, 2014). However, the addition of SPS cue re-exposure during OFT and SMT did not enhance or unmask changes in this study. Cue re-exposure during the OFT did not induce group differences in anxiety-like behaviors in SPS rats, suggesting re-exposure was not sufficient to trigger expected phenotypes. This was also noted in the SMT, as cue re-exposure did not alter motivation for novel social and novel object interactions. The lack of cue-driven behavioral changes in the SPS group was unexpected in this study and suggests that the SPS model (i.e., US) was not sufficiently aversive to create a robust fear memory associated with the paired neutral odor (i.e., CS).

Although only male rats were initially assessed for changes without cue re-exposure, both females and males were included in the investigation of SPS cue re-exposure during OFT and SMT. This limitation prevented direct comparison of males and females outside of this group, and sample sizes were not adequately powered for sex-based analyses in the cue re-exposure group. The overall lack of behavioral phenotypes in either condition following SPS further limits the evaluation of any sex differences that may exist when outcomes are more robust. A much larger sample size may be required to detect subtle effects in OFT or SMT in either males or females. Although these tasks were chosen to assess general locomotion, anxiety-like behavior, and social motivation or avoidance, alternative behavioral tasks may provide more sensitive readouts of stress-induced phenotypes.

### CORT levels were unaltered at 1 and 2 weeks following SPS

There were no significant group changes in serum CORT levels 1 or 2 weeks following SPS in rats. Additionally, there was no evidence of CORT changes (i.e., pre to post SPS) that aligned with behavioral phenotypes. A reduced baseline cortisol level (i.e., hypocortisolism) has been commonly related to PTSD, demonstrating HPA axis dysregulation even without restress (von Majewski et al., 2023). Decreased baseline CORT has been reported following SPS in rats, although not until 28 d post-SPS (Zhang et al., 2012). Our lack of CORT signal, in the absence of a restress event, may suggest an inadequate physiological stress from SPS that aligns with the null behavioral findings. The timepoints of CORT sampling were chosen to put potential HPA axis changes, and their timing, in the context of behavioral assessments. However, the lack of an acute sampling (e.g., within hours or days of SPS) limits interpretability and leaves open the possibility that our SPS model did not elicit the acute stress response that has been reported following the individual components of SPS (Abel, 1993; Zardooz et al., 2010; Bekhbat et al., 2018) and contributed to our negative results. It is also possible that a dexamethasone suppression test would have provided an alternate approach to assess HPA axis functionality. Methodological choices, such as the use of isoflurane during serum collection, were made to reduce variation and maximize potential group comparisons. It is unlikely the brief use of isoflurane would have significantly altered CORT levels. Previous studies have shown little to no effect following brief exposure in males, although some evidence suggests more female sensitivity (Zardooz et al., 2010; Bekhbat et al., 2016).

### High individual and group variation across metrics

Individual behavioral and CORT variation was assessed relative to the mean control group performance to determine the extent of variation in the groups and to identify potential deficits across metrics per animal. Further, this assessment was performed to identify potential subgroups of SPS rats or if susceptibility and deficits only occurred in some individuals. No evidence of latent SPS subgroups emerged from this question, as both groups had very few individual animal *Z*-scores that were significantly different from controls. Approximately 5% of comparisons were significant, as would be expected by chance. Additionally, individual SPS rats did not have significant performance differences from controls in multiple metrics, whether behavioral or CORT-related, and individual variation was not altered by the presence cue re-exposure during behavior. In sum, this confirms that despite SPS model induction, there were no substantial or consistent changes in behavioral performance or CORT levels.

### Considerations for the future of the SPS model

The SPS model has been widely used and published to study PTSD related behavioral outcomes in rodents, but the consistency and validity of this model are still subject to debate. SPS is intended to produce phenotypes relevant to individuals with PTSD, but many diagnostic criteria are subjective or overlooked (e.g., criteria of duration in PTSD) and limit translatability when designing experiments. Small procedural changes can also strongly influence the presence or absence of group differences, so comparison across studies remains difficult (Ferland-Beckham et al., 2021). Additionally, there are limited reports addressing the SPS model in female rats, with mixed results depending on behavior and timeline (Keller et al., 2015; Pooley et al., 2018a,b; Nahvi et al., 2019; Mancini et al., 2021). Evidence that SPS in females may not reliably induce deficits related to PTSD continues to emerge, which limits translational value.

Factors that divide studies into “produced phenotype” versus “no phenotype” may come down to minor procedural differences, such as individual versus group exposure to stressors (Vanderheyden et al., 2015) or degree of isolation during quiescence week. This suggests that the model lacks robustness to produce consistent behavioral changes across research groups. Some studies chose to combine SPS with additional stress-related paradigms like contextual fear conditioning to demonstrate enhanced fear memory (Kohda et al., 2007) or have switched to alternative models to enhance the robustness of phenotypes in certain assays (Skórzewska et al., 2020; Pitcairn et al., 2024). Future research should focus on maximizing reliability and consistency of outcomes, particularly in producing robust trauma-induced phenotypes, which may require modifications to the SPS model, testing alternate behaviors, or consideration of timepoints.

### Conclusions

In this study, SPS did not produce behavioral phenotypes in anxiety-like or social domains, or evidence of dysregulated HPA axis function at 1 or 2 weeks in rats. Cue re-exposure did not enhance weak behavioral outcomes, showing SPS also failed to elicit trauma-cue avoidance. The protocol used for this study did not appear to induce sufficient stress to produce robust, expected phenotypes at a group level. These null findings differ from reported literature, where SPS has largely been upheld as a common model for studying behavioral and physiological changes relevant to PTSD. The lack of a robust behavioral effect limits the ability to assess temporal changes or potential novel therapies. We chose not to employ a modified SPS protocol, as this paradigm has been reported as sufficient to produce behavioral and CORT changes 1 week post-SPS. This SPS model may produce some acute deficits (i.e., <1 week), but acute or temporary changes may not relate to symptoms and dysregulation in individuals with PTSD. It is well understood that all preclinical models have inherent limitations, but findings suggest that this induction protocol for SPS, without modification or enhancement, lacks robustness and replicability for continued use and consideration as a good model for translational PTSD studies.

## Synthesis

Reviewing Editor: Mihaela Iordanova, Concordia University - Loyola Campus Decisions are customarily a result of the Reviewing Editor and the peer reviewers coming together and discussing their recommendations until a consensus is reached. When revisions are invited, a fact-based synthesis statement explaining their decision and outlining what is needed to prepare a revision will be listed below. The following reviewer(s) agreed to reveal their identity: NONE.

The reviewers found the manuscript to be well-written, easy to follow and the question under study of significant importance. The reviewers raised a number of concerns that would greatly strengthen the manuscript if addressed.

Major, to be addressed.

1) Effectiveness of the SPS. With any negative results, a question that arises is whether a procedure was appropriate conducted. To ensure that the negative results are meaningful, some evidence of the effectiveness of the SPS is needed. One reviewer suggested the assessment of plasma CORT levels immediately following SPS cessation. This would then suggest that the SPS model is sufficient at producing physiological alterations, but that these changes are not persistent or long-lasting.

2) Methodological details. Several methodological details are missing, which limit the interpretability of the results. For example, the history and characteristics of the stimulus rodents used in the social interaction tasks are not described. The rationale for the selected timepoints for corticosterone collection, as well as the decision to perform sampling under anesthesia (which may itself induce stress), should be clarified. Additionally, the sex distribution is highly unbalanced, with disproportionately fewer female rats, and the reasons for this discrepancy should be addressed or additional data collected.

3) Figures. Figures should also be revised-currently, they are small and difficult to interpret. Distinct shapes or colors should be used to clearly differentiate male and female data points. If Figure 4 presents data from a different cohort, the authors should apply the same approach across all figures for consistency, or alternatively combine cohorts where appropriate. Figure 5 is particularly hard to read and should be revised for clarity.

4) Discussion. The discussion is overly long and occasionally speculative and redundant. A more concise and focused discussion would improve readability and strengthen the manuscript.

Minor, left to the discretion of the authors.

5) A demonstration that the SPS protocol used can enhance fear memory as previously reported in the literature (Kohda et al., 2007) or employing alternative anxiety-related tasks, may also help in interpreting the effectiveness of SPS and thus strengthen the negative results.

6) Is it possible that brief exposure to the isofluorane anesthesia at the time when the blood samples are collected could be affecting corticosterone levels and masking any differences between groups compared to if no-anesthesia was used?

7) To strengthen the study's impact the authors may want to consider offering a new conceptual approach or theoretical framework. The authors could provide a clearer rationale and broader context for their findings, situating this work within a larger framework of stress research and PTSD modeling.

## Author Response

Response to Review, eNeuro eN-NRS-0168-25R1 11-20-2025 Manuscript Title: "The single-prolonged stress model fails to produce behavioral or corticosterone alterations in rats" Dear Dr. Iordanova, Thank you for the opportunity to revise and resubmit our manuscript to eNeuro. We appreciate the thoughtful feedback provided by you and the reviewers. We have carefully addressed the two remaining minor concerns, improving the clarity of the manuscript. Below we have outlined the minor points raised and the revisions made, which are shown highlighted in the revised manuscript. We believe that these revisions have addressed the minor concerns regarding the discussion and improved the specificity and conciseness of the manuscript, particularly in previously revised text.

Minor Revisions Minor Points 1) Clarification in the Discussion that the lack of confirmation of acute increases in CORT following SPS leaves open the possibility that SPS was not be effective in increasing CORT levels and as a result may not have been effective in driving differences between the groups.

We appreciate the feedback on this section and have revised the Discussion to more clearly explain. We hope that the edits to the statement and surrounding paragraph better convey the point surrounding CORT results and interpretation. As described in the Methods, serum collection timepoints were chosen to align with our aim of assessing enduring, post-isolation effects. No samples were obtained within hours or days of SPS completion- however, the text more clearly explains that individual components of SPS (i.e., restraint, diethyl-ether) have been reported to cause an acute CORT increase. Because we did not perform an acute sample following the multi-stressor SPS protocol, our data cannot distinguish whether (a) an acute CORT elevation occurred and then normalized prior to one week post-SPS, or (b) the SPS protocol did not elicit a substantial acute CORT change. In addition to addressing this point directly in the text, we revised the surrounding Discussion paragraph to improve clarity of interpretation, relationship to previous literature, and previously unclear limitations.

2) In many places the revised text lacked clarity or was grammatically awkward. A few examples of this are included below but a closer look at Ms is needed and appropriate revision must be made. • The second sentence of the abstract spans 6 lines (Line 5-11) and it is hard to follow. Moreover, it's is unclear what 'behavioural domains' refers to. Such vague language is not helpful in scientific writing. • Relatedly, the use of 'domains' in the sentence 'Many individuals with PTSD experience limited relief to symptom domains ...' (line 37) seems unnecessarily confusing. • Line 338-343 also suffers from lack of clarity due, in part, to the length of the sentence. Line 344. The sentence 'Both females and males were included in the investigation of cue re-exposure, but otherwise behavior was in male rats.' is poorly constructed and lacks specificity.

We thank the reviewers for highlighting areas where clarity and conciseness should be improved, as well as the suggesting a thorough review of text for readability. In response, we have addressed the lines of concern, as well as conducted a general review for grammar and appropriate revisions. As noted by the reviewers, portions of the abstract and manuscript were difficult to follow due to long, spanning sentences and vague language. The abstract, including the long second sentence noted by reviewers, has been rewritten more concisely. Unnecessarily long and unclear sentences in other sections, including portions of the Introduction and Discussion, were similarly modified to improve readability and align with reviewer feedback.

Additionally, we removed vague terminology including "behavioral domains" from the abstract and main text, instead clearly defining the behavioral outcomes. This replaced unclear statements surrounding domains with more specific language surrounding anxiety-like and social behaviors that better inform the manuscript. The sentence referring to "symptom domains" in individuals with PTSD has been restructured to remove unnecessary abstraction and improve readability. Additionally, the paragraph introducing PTSD and the rationale for preclinical models has been reorganized so that the role of rodent models is more clearly established (prior to clinical context).

The reviewers also identified examples of unclear or awkward phrasing in the Discussion. These lines, as well as the discussion, was simplified of unnecessarily long or confusing sentences. For example, the sentence 'Both females and males were included in the investigation of cue re-exposure, but otherwise behavior was in male rats' has been replaced with a clearer and more precise explanation of the sex composition, and the implications for result interpretation. Beyond the specific incidences shared by the reviewers, we did a comprehensive revision of the manuscript as suggested to improve clarity, grammar, and readability. Additional sentences or sections were edited to align with the suggestions provided, resulting in enhanced specificity, reduced vague and abstract language, and more clarity throughout the manuscript.

Final Remarks We appreciate the careful attention to the clarity and readability of our manuscript by the reviewers, and for the opportunity to revise our submission based on your feedback. We believe that these revisions responding to the two minor points have improved the quality of the submission. We hope that the changes adequately address all the remaining concerns, and we would like to thank you for all your time and considerations surrounding the publication of our manuscript.

Sincerely, Corresponding Author on behalf of all co-authors Previous Minor Revisions eN-NRS-0168-25R (submitted 08-2025) Major Points 1) Effectiveness of the SPS. With any negative results, a question that arises is whether a procedure was appropriately conducted. To ensure that the negative results are meaningful, some evidence of the effectiveness of the SPS is needed. One reviewer suggested the assessment of plasma CORT levels immediately following SPS cessation. This would then suggest that the SPS model is sufficient at producing physiological alterations, but that these changes are not persistent or long-lasting.

We appreciate this important point regarding interpretation of null findings. While we did not include a separate cohort for acute corticosterone (CORT) sampling immediately after SPS and acknowledge the concern for procedural variation, we designed the study to evaluate whether more chronic behavioral and physiological changes were present one or two weeks post-induction, intentionally beginning after the isolation period that is part of the classic SPS model (Liberzon et al., 1997). We revised the Discussion to articulate the choice of timeline, including that the SPS model has been reported to induce CORT dysregulation at or after the 1-week mark, and that our sampling timepoints were chosen based on this established literature (e.g., Liberzon et al., 1997; Zhang et al., 2012). Although some studies (Kohda et al. 2007) demonstrate acute increases in plasma CORT, these normalize within hours to one day, which may not translate to chronic stress-related phenotypes relevant to PTSD, which has a necessary criteria of duration following trauma.

To reinforce this point of acute stressor changes in the hours following SPS, we have added language noting that individual SPS components (e.g., forced swim, diethyl ether) are known to acutely elevate CORT (Abel, 1993; Bekhbat et al., 2018, Zardooz et al. 2010), but such transient responses do not reflect the chronic dysregulation expected in translational PTSD models. We discuss that CORT alterations in the hours post-SPS would likely reflect stress from diethyl ether exposure and would not clarify whether long-term HPA-axis changes occur.

2) Methodological details. Several methodological details are missing, which limit the interpretability of the results. For example, the history and characteristics of the stimulus rodents used in the social interaction tasks are not described. The rationale for the selected timepoints for corticosterone collection, as well as the decision to perform sampling under anesthesia (which may itself induce stress), should be clarified. Additionally, the sex distribution is highly unbalanced, with disproportionately fewer female rats, and the reasons for this discrepancy should be addressed or additional data collected.

We thank the reviewers for these important methodological suggestions. In the revised Methods and Discussion, we have addressed each of these points:

Novel Object and Social Stimulus in SMT: We specify that the social stimulus rats were sex- and age-matched, novel conspecifics and provide additional methodological detail for the novel objects used in each SMT session.

Timepoints for CORT Sampling: A detailed explanation was added to the Methods > CORT Collection and Discussion, clarifying that blood was drawn at baseline, one week (end of isolation), and two weeks post-SPS to assess post-induction physiological changes aligned with expected timing from prior studies (Liberzon et al., 1997; Zhang et al., 2012). This reflects our aim to assess enduring rather than acute effects, which would have occurred during the isolation window.

Use of Isoflurane for Blood Sampling: We added justification in the Methods and Discussion that brief isoflurane exposure was used to minimize distress and facilitate reliable sampling from the lateral tail vein. Supporting literature (e.g., Bekhbat et al., 2016; Zardooz et al., 2010) suggests that brief isoflurane anesthesia does not significantly affect CORT levels in male rats, though we acknowledge potential female sensitivity. However, no sex-based CORT differences were detected in our study.

Sex Distribution: We explain that the study was initially designed to ensure replication of prior findings in males prior to advancing to females, given that most SPS literature is male-focused. After observing null results in males, we included a separate study of females and males to test cue re-exposure effects, in attempts of enhancing or re-invigorating phenotypes. Although not powered for sex comparisons, no sex differences were detected, and sexes were combined for relevant analyses. We also cite reports showing mixed findings with SPS in females, reinforcing that this model may not reliably produce sex-specific phenotypes. We acknowledge that we are limited in our comparisons, although a putative model for SPS should produce robust phenotypes Irregardless of sex, although sex-differences may emerge.

3) Figures. Figures should also be revised-currently, they are small and difficult to interpret. Distinct shapes or colors should be used to clearly differentiate male and female data points. If Figure 4 presents data from a different cohort, the authors should apply the same approach across all figures for consistency or alternatively combine cohorts where appropriate. Figure 5 is particularly hard to read and should be revised for clarity.

We appreciate the feedback regarding figure clarity. All figures have been thoroughly revised:

Figure resizing: All figures were reformatted to comply with eNeuro's guidelines and are now publication-quality EPS files (300 dpi) at appropriate column dimensions.

Sex identification: Male and female data points are now shown with distinct shapes and/or colors in all relevant figures.

Legends updated: Sex labels ("M" and "F") have been added to all relevant figure legends.

Figure 4: Data were combined across cohorts to increase interpretability, while also displaying individual sexes using distinct markers.

Figure 5: The layout, font size, contrast, and labeling were improved for clarity. Individual animal IDs include sex designation.

4) Discussion. The discussion is overly long and occasionally speculative and redundant. A more concise and focused discussion would improve readability and strengthen the manuscript.

We appreciate the reviewer's comment and have undertaken a careful revision of the Discussion to improve focus, remove redundancy, and eliminate speculative language. Specifically, we removed repetitive phrasing that reiterated the null findings without adding interpretation. A more thorough rationale of timepoints and methodological choices, and their relationship to other findings, was provided to situate our intention and results compared to other studies. We revised the Considerations for the Future of the SPS Model section to eliminate conjecture and focus on the major translational limitations of the model and optimizations (modified SPS or alternate PTSD model) for testing chronic trauma-induced phenotypes. Together, these edits reduce length, remove speculation, and sharpen the manuscript's critical evaluation of SPS as a preclinical PTSD model.

Minor Points (Author Discretion) 5) A demonstration that the SPS protocol used can enhance fear memory as previously reported in the literature (Kohda et al., 2007) or employing alternative anxiety-related tasks, may also help in interpreting the effectiveness of SPS and thus strengthen the negative results.

We appreciate the reviewer's suggestion and have addressed this point by expanding the introduction and discussion to include a consideration of studies such as Kohda et al. (2007), which have shown that the SPS model can lead to enhanced fear memory when paired with contextual fear conditioning (CFC). However, we note that such findings typically emerge in protocols where CFC is introduced as an additional stressor after SPS, creating a compound model rather than assessing SPS effects alone. Our study intentionally sought to evaluate the ability of SPS, as it is most commonly implemented, to independently produce persistent behavioral and physiological alterations.

The need to pair SPS with additional stress paradigms in order to yield positive findings, in this case in the fear domain, calls into question whether SPS alone is sufficient as a robust model for PTSD-like phenotypes. Clarification to this information is now included in the introduction to provide broader context and again in the discussion to highlight that while such combined paradigms may be effective in producing certain behavioral outcomes, they diverge from traditional SPS-alone models. We argue that the absence of long-term phenotypes in our study underscores an important limitation of SPS as a standalone model.

6) Is it possible that brief exposure to the isofluorane anesthesia at the time when the blood samples are collected could be affecting corticosterone levels and masking any differences between groups compared to if no-anesthesia was used? This is a valid consideration, and we have addressed it in the revised methods and discussion sections. Our protocol used brief isoflurane anesthesia to allow for rapid and consistent blood collection from the lateral tail vein, which was necessary to obtain sufficient volume for serum collection and replicates in ELISA analyses. Although it is true that anesthetic agents may induce stress or alter corticosterone levels under some conditions, exposure times were brief according to prior research demonstrating that brief isoflurane exposure does not significantly impact CORT levels in male rats (e.g., Bekhbat et al., 2016), although limited studies have shown more impact in females. We did not observe evidence of a CORT effect driven by sex in our data, suggesting that this method did not introduce systematic bias across groups. While we acknowledge the limitation and possibility of subtle effects, the use of isoflurane in this context represents a reasonable and common compromise between minimizing stress and ensuring reliable sample collection. This rationale is now included in the manuscript to clarify our methodological choices and their implications.

7) To strengthen the study's impact the authors may want to consider offering a new conceptual approach or theoretical framework. The authors could provide a clearer rationale and broader context for their findings, situating this work within a larger framework of stress research and PTSD modeling.

We appreciate this thoughtful suggestion and have modified the manuscript in hopes of more clearly situating our approach and to establish our findings in the field. We better describe that study was designed to test whether the SPS model, widely cited as a preclinical model for PTSD, produces persistent and robust behavioral and physiological outcomes at more chronic (one and two weeks) timepoints, and whether SPS could be paired with cue re-exposure to enhance chronic phenotypes. This inquiry aligns with growing concerns in the literature regarding the variability and replicability of SPS-induced phenotypes across laboratories, particularly when it comes to long-term effects and translational value for PTSD research. Our revised text emphasizes this broader conversation, highlighting that a valid translational model should generate robust, long-term phenotypes without requiring additional modifications or stressors. By articulating these concerns, we hope to contribute constructively to ongoing efforts to refine or re-evaluate the effectiveness of models used to study chronic stress and trauma-related disorders.
